# Healthcare Workers Who Work With COVID-19 Patients Are More Physically Exhausted and Have More Sleep Problems

**DOI:** 10.3389/fpsyg.2020.625626

**Published:** 2021-01-08

**Authors:** Henrico van Roekel, Irene M. J. van der Fels, Arnold B. Bakker, Lars G. Tummers

**Affiliations:** ^1^Utrecht School of Governance, Utrecht University, Utrecht, Netherlands; ^2^IZZ, Apeldoorn, Netherlands; ^3^Center of Excellence for Positive Organizational Psychology, Erasmus University Rotterdam, Rotterdam, Netherlands

**Keywords:** COVID-19, healthcare workers, physical exhaustion, mental exhaustion, sleep problems, general health

## Abstract

In this survey study of 7,208 Dutch healthcare workers, we investigate whether healthcare workers dealing with COVID-19 patients experience lower general health, more physical and mental exhaustion and more sleep problems than other healthcare workers. Additionally, we study whether there are differences in well-being within the group of healthcare workers working with COVID-19 patients, based on personal and work characteristics. We find healthcare workers who are in direct contact with COVID-19 patients report more sleep problems and are more physically exhausted than those who are not in direct contact with COVID-19 patients. Mental exhaustion and general health do not significantly differ between healthcare workers who are in direct contact with COVID-19 patients and those who are not. Among healthcare workers in direct contact with COVID-19 patients, lower well-being on one or more indicators is reported by those who are female, living alone, without leadership role, or without sufficient protective equipment. Regarding age, physical exhaustion is more prevalent under healthcare workers older than 55 years, whereas mental exhaustion is more prevalent under healthcare workers younger than 36 years. These results stress the need of mental and physical support of healthcare workers during a pandemic, catered to the needs of healthcare workers themselves.

## Introduction

The COVID-19 pandemic has presented great threats to the well-being of healthcare workers. Many of them risked infection with the virus while working longer hours in understaffed organizations (Adams and Walls, 2020; Mhango et al., 2020; Pearman et al., 2020; Wang et al., 2020). Since the outbreak, scholars have presented first results on what effects the crisis has had on healthcare workers. Studies show effects on attitudes and practices, like a high fear of self-infection (Zhou et al., 2020), an increase in mental health problems like job stress and anxiety (Cao et al., 2020; Spoorthy et al., 2020; Tan et al., 2020; Wei et al., 2020), and the development of physical problems like increased headaches due to wearing protective equipment (Ong et al., 2020). Similarly, a scoping review of 37 studies on how COVID-19 has impacted healthcare worker wellness showed COVID-19 was associated with, among else, more stress, anxiety and poorer quality of sleep (Shreffler et al., 2020).

However, we know little about whether the effects of the COVID-19 outbreak on healthcare workers’ well-being differ across groups of healthcare workers. We therefore firstly study whether healthcare workers dealing with COVID-19 patients experience more threats to well-being than other healthcare workers. For instance, is it truly the case that healthcare workers working with COVID-19 patients report more exhaustion? Second, we study whether there are differences within the group of healthcare workers who work with COVID-19 patients. Besides, studies on healthcare worker well-being are mainly conducted in Asian context (Cao et al., 2020; Shreffler et al., 2020; Spoorthy et al., 2020; Tan et al., 2020; Zhou et al., 2020). We present data on Dutch healthcare workers to address these gaps.

As healthy healthcare workers are crucial in the aftermath of the outbreak, and in prevention of further outbreaks, losing a substantial part of the workforce to psychological or physical threats is detrimental. Therefore, the results can fuel healthcare organization policies and human resource practices to sustain the mental and physical health of healthcare workers during and after COVID-19.

## Methods

We collected data in a May-June 2020 cross-sectional survey on work and health of Dutch healthcare workers. Healthcare workers were invited *via* email to voluntarily participate in the online survey and they were reminded after a few weeks. To protect their identities, respondents were not asked to give their names and contact information; other potentially identifiable data, such as gender, age, and job type were carefully protected. A total of 7,208 respondents completed our survey. Data used in this article is included as an Supplementary Material.

We use four employee well-being measures as dependent variables: a general health measure asking respondents to rate their general health [10-point scale ranging from 1 to 10 (Sullivan and Karlsson, 1998)], mental exhaustion [five items on a 5-point Likert scales ranging from 1 (never) to 5 (always) (daily), example item: I feel mentally exhausted because of my work (Schaufeli, 1996)], physical exhaustion [five items on a 5-point Likert scales ranging from 1 (never) to 5 (always) (daily), example item: I feel physically exhausted because of my work (Schaufeli, 1996)], and sleep problems [three items on a 5-point Likert scales ranging from 1 (no) to 5 (a lot), example item: I have a restless or disturbed sleep (Adriaenssens et al., 2012)].

For our independent variables, we compare the well-being outcomes between groups based on personal and work characteristics. First, we assess whether outcomes differ for healthcare workers who do and do not work in direct contact with COVID-19 patients. Next, within the group of healthcare workers who work with COVID-19 patients, we assess multiple variables to define risk groups of healthcare workers. To do so, we study three personal characteristics: gender, age, and whether the healthcare worker lives alone. For age, we divide our sample into three categories: younger than 36, between 36 and 55, and older than 55. This is a common division of younger, middle-aged and older employees used in academic research as well as governmental research on well-being. It also enables to assess non-linear relationships with well-being. Additionally, we study two important work characteristics: leadership role (whether the healthcare worker indicates to have a leadership role) and sufficient protective equipment (healthcare workers were asked: “do you have sufficient protective equipment at your disposal?”; they could answer with yes or no). In selecting these variables, we have not aimed to be exhaustive, but to constitute a broad picture of factors potentially related to well-being.

Our sample (*N* = 7,208) is representative for Dutch healthcare workers in terms of gender: our sample has 82% females, while for Dutch healthcare workers this is 84%. However, our sample is older (*x̄* = 51.5 versus *x̄* = 42.5) (CBS data from^1^). Furthermore, our respondents represent all healthcare industries: hospitals (36.2%), nursing homes and homecare (23.6%), mental health care (16.5%), disability care (17%) and other healthcare industries (6.7%).

For analyses, we conduct t-tests or ANOVA’s, when appropriate. For the ANOVA’s we conduct *post hoc* analyses (Tukey’s HSD) to define which groups significantly differ. The level of significance is set at 0.05 and Cohen’s *d* effect sizes are calculated (Cohen, 1988). Secondly, as additional analysis, multivariate regression analyses are performed for each of the four well-being variables to gain more understanding on the relative strength with which the variables are related to well-being. The defined groups are included as independent variables. We report adjusted R-squared values for the models and Beta-values to indicate the relative strength of each variable.

## Results

We start by contrasting healthcare workers who work in direct contact with COVID-19 patients versus those who do not (Table 1). Healthcare workers in direct contact with COVID-19 patients report significantly more sleep problems and physical exhaustion. No significant differences are found for mental exhaustion or general health.

**TABLE 1:** More sleep problems and physical exhaustion for healthcare workers in direct contact with COVID-19 patients.

Direct contact COVID-19 patients		Sleep problems			Physical exhaustion			Mental exhaustion			General health		
	*N*	*x̄* (SD)	*t*(7,206)	*d*	*x̄* (SD)	*t*(7,206)	*d*	*x̄* (SD)	t(7,206)	*d*	*x̄* (SD)	*t*(7,206)	*d*
Yes	2,621	2.42 (0.97)	−6.45**	0.159	2.29 (0.82)	−9.21**	0.223	1.98 (0.76)	−0.64	−	7.61 (1.26)	−1.49	−
No	4,587	2.27 (0.91)			2.11 (0.79)			1.97 (0.74)			7.56 (1.31)		

Cohen’s d effect sizes are small (Cohen, 1988). **<0.01.

Next, we zoom in within the group of healthcare workers in direct contact with COVID-19 patients (Table 2). First, female healthcare workers report more sleep problems and physical exhaustion than male healthcare workers, whilst there are no significant differences on mental exhaustion and general health.

**TABLE 2:** Differences within the group of healthcare workers who are in direct contact with COVID-19 patients.

		Sleep problems			Physical exhaustion			Mental exhaustion			General health		
	*N*	*x̄* (SD)	*t*(2,619)	Cohen’s *d*	*x̄* (SD)	*t*(2,619)	Cohen’s *d*	*x̄* (SD)	*t*(2,619)	Cohen’s *d*	*x̄* (SD)	*t*(2,619)	Cohen’s *d*
**Gender**													
Female	2,201	2.46 (0.97)	4.73**	0.226	2.31 (0.81)	2.73*	0.145	1.98 (0.76)	0.41	–	7.60 (1.24)	−0.59	–
Male	420	2.21 (0.91)			2.19 (0.84)			1.96 (0.77)			7.64 (1.40)		
**Age**													
<36 years	277	2.36 (0.96)	1.09^*c*^	–	2.30 (0.80)	6.77**^c^		2.10 (0.83) ^	4.41*^c^	0.191^*b*^	7.61 (1.21)	0.49^*c*^	–
36–55 years	1,347	2.41 (0.97)			2.23 (0.79)			1.95 (0.74)			7.63 (1.26)		
>55 years	997	2.45 (0.96)			2.36 (0.86)^∧^		0.157^*a*^	1.98 (0.78)			7.58 (1.29)		
**Living alone**													
No	2,151	2.41 (0.97)	−1.24	–	2.26 (0.81)	−3.12**	0.158	1.96 (0.75)	−3.18**	0.155	7.64 (1.25)	2.20*	0.112
Yes	470	2.47 (0.95)			2.39 (0.84)			2.08 (0.80)			7.49 (1.32)		
**Leadership role**													
No	2,199	2.43 (0.96)	1.20	–	2.30 (0.82)	2.51*	0.121	1.98 (0.76)	0.36	–	7.59 (1.26)	−1.75	–
Yes	422	2.37 (0.99)			2.20 (0.83)			1.97 (0.77)			7.71 (1.31)		
**Sufficient protective equipment**													
No	507	2.67 (1.03)	−6.67**	0.314	2.60 (0.89)	−9.90**	0.466	2.33 (0.84)	−11.98**	0.562	7.24 (1.40)	7.48**	0.352
Yes	2,114	2.36 (0.94)			2.21 (0.78)			1.89 (0.72)			7.70 (1.21)		

Cohen’s d effect sizes are small to medium (Cohen, 1988). *p < 0.05; **p < 0.01. ^∧^, significantly different from healthcare workers between 36 and 55 years; a, effect size between age categories 36–55 years and >55 years; b, effect size between age categories <36 years and 36–55 years; c, *F*-test (df = 2,618).

Regarding healthcare workers’ age, physical exhaustion is more prevalent among healthcare workers who are older than 55 compared to healthcare workers between 36 and 55 years old. In contrast, mental exhaustion is more prevalent among healthcare workers who are younger than 36, compared to healthcare workers between 36 and 55 years old. There are no significant differences between age categories on sleep problems and general health.

Additionally, we assess whether living alone or with family is correlated with well-being. We find that healthcare workers who live alone report higher physical and mental exhaustion and lower general health. No significant differences are found for sleep problems.

Next, we consider work characteristics. Healthcare workers without a leadership role are found to be more physically exhausted than healthcare workers who have a leadership role. No significant differences of having a leadership role are found for sleep problems, mental exhaustion and general health.

Finally, is having sufficient protective equipment in working with COVID-19 patients correlated with well-being? We find significant differences for all outcomes: healthcare workers who do not have sufficient protective equipment report more sleep problems, more physical and mental exhaustion, and lower general health.

In additional analysis we conduct multivariate regression analyses per well-being outcome. The analyses yield similar results as above. For sleep problems, gender [reference = female; β = −0.09, *t*(2,614) = −4.49, *p* < 0.05] and having sufficient protective equipment [ref. = sufficient equipment; β = 0.12, *t*(2,614) = 6.35, *p* < 0.05] are significant predictors (Adj. *R*^2^ = 0.024). For physical exhaustion, gender [β = −0.05, *t*(2,614) = −2.40, *p* < 0.05], living alone [ref. = not living alone; β = 0.05, *t*(2,614) = 2.49, *p* < 0.05], being older than 55 [β = 0.07, *t*(2,614) = 3.61, *p* < 0.05], leadership role [ref. = no leadership role; β = −0.04, *t*(2,614) = −2.11, *p* < 0.05], and having sufficient protective equipment [β = 0.19, *t*(2,614) = 9.66, *p* < 0.05] are significant predictors (Adj. *R*^2^ = 0.045). For mental exhaustion, living alone [β = 0.05, *t*(2,614) = 2.83, *p* < 0.05], being younger than 36 [β = 0.06, *t*(2,614) = 2.80, *p* < 0.05], and having sufficient protective equipment [β = 0.23, *t*(2,614) = 11.97, *p* < 0.05] are significant predictors (Adj. *R*^2^ = 0.056). Finally, for general health, living alone [β = −0.04, *t*(2,614) = −1.97, *p* < 0.05] and having sufficient protective equipment [β = −0.14, *t*(2,614) = −7.40, *p* < 0.05] are significant predictors (Adj. *R*^2^ = 0.021).

## Discussion

In this brief research report we have investigated whether healthcare employees who work with COVID-19 patients report lower wellbeing and whether differences exist within that group. Our results confirm that healthcare workers who treat COVID-19 patients experience more sleep problems and physical exhaustion compared to healthcare workers who do not treat COVID-19 patients. Furthermore, some personal and work characteristics present higher well-being risks.

In the light of the extant literature it should be acknowledged that in our study, effects are small to medium. In some of the other contexts, wellbeing appears to have decreased more drastically (Shreffler et al., 2020). What is more, mean scores still appear relatively acceptable (e.g., the lowest group score for general health is 7.24). This may point to the fact that the Netherlands has a relatively well organized healthcare system (Daley et al., 2013). Nevertheless, our study contributes to the literature by, firstly, comparing effects across groups of healthcare workers, and secondly, presenting data from a non-Asian context.

There are a few limitations to discuss. First, whilst we employ validated scales, due to practical constraints in executing our survey we were not able to use clinical validated scales. Second, our data is collected in May-June 2020, right after the “first peak,” and the COVID-19 crisis as well as effects on well-being have developed since. Similarly, our cross-sectional design limits causal inference. Ergo, future research can improve on our current design by using validated tests, and employing longitudinal designs to track healthcare worker well-being over time.

Considering the practical implications of our study, we urge healthcare leaders, managers, and HR professionals to maintain healthcare worker well-being. Whilst a pandemic is hard to control, there are best practices on how to help healthcare workers deal with the consequences through, for example, job redesign, counseling, a behavioral health hotline, stress management webinars, respite rooms and creating celebratory rituals (Wei et al., 2020). Herein, our results show healthcare leaders should pay special attention to the groups of healthcare workers who appear disproportionally affected regarding either general health, physical or mental well-being, or sleep. Additionally, our results may fuel a number of questions to be discussed. For example, should more vulnerable healthcare workers (e.g., elderly female) be less actively deployed among COVID-19 patients? Which job resources help healthcare workers to deal with COVID-19 stressors including threats of infection, insecurity, work pressure, emotional demands, and work-family conflict (Foley et al., 2020; Kniffin et al., 2020)? How can healthcare workers be stimulated to share leadership to actively improve their own working conditions? The results also emphasize the grave importance of sufficient protective equipment. In conclusion, healthcare leaders are required to actively anticipate the evolution of this pandemic in order to maintain healthcare worker well-being; studies like these may help them to do just that (Torbay, 2020).

## Data Availability Statement

The original contributions presented in the study are included as Supplementary Material, further inquiries can be directed to the corresponding author.

## Ethics Statement

The studies involving human participants were reviewed and approved by the Faculty Ethical Review Committee of Utrecht University. The participants provided their written informed consent to participate in this study.

## Author Contributions

All authors contributed to the article and approved the submitted version.

## Conflict of Interest

The authors declare that the research was conducted in the absence of any commercial or financial relationships that could be construed as a potential conflict of interest.

## Footnotes

1 https://azwstatline.cbs.nl
